# Supplementary material for: Mass Spectra Fitting as Diagnostic Tool for Magnetron Plasmas Generated in Ar and Ar/H2 Gases with Tungsten Targets
Source: Molecules. 2023 Jul 26;28(15):5664. doi: 10.3390/molecules28155664 (PMC10420293; doi:10.3390/molecules28155664)
Supplement: Supplementary file 1 [file molecules-28-05664-s001.zip › molecules-2488342-supplementary.pdf]

Supplementary Material

Peaks presenting the asymmetric and trapezoidal shape characteristic for our mass spectra are shown In Figure S1: a) Cu^{+}, b) $Ag^{+,}$ c) Au^{+}, d) Th^{+} species.

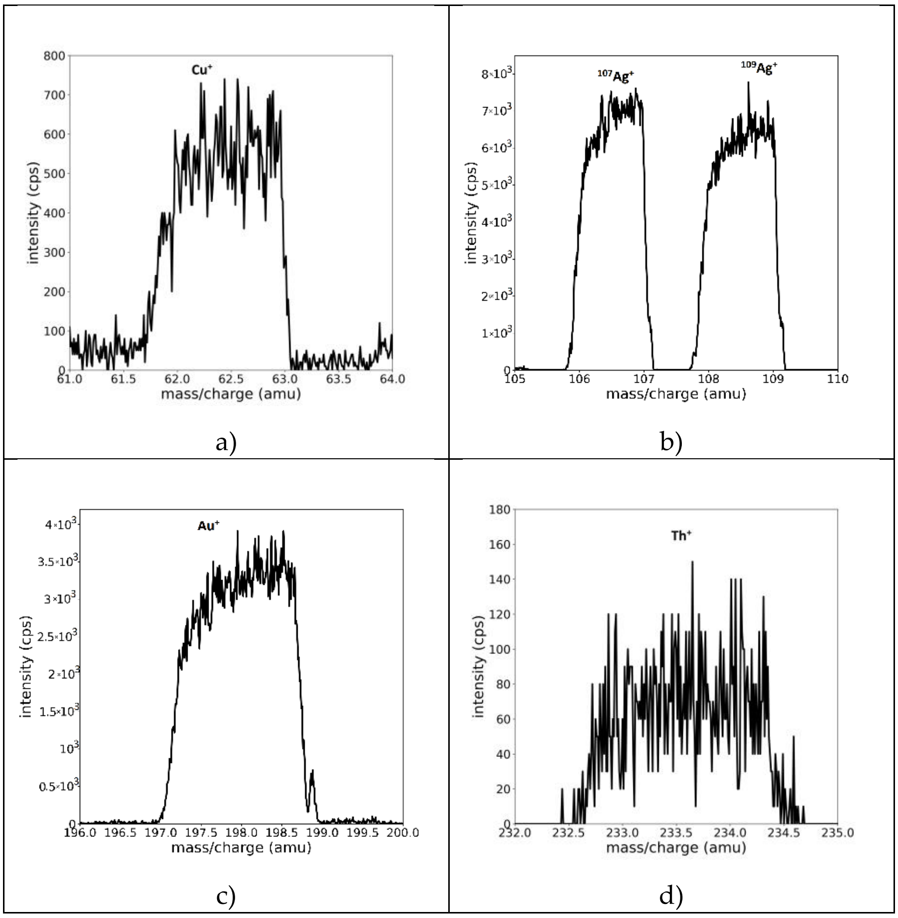

Figure S1. Well isolated peak profiles, presenting the asymmetric flat/trapezoidal shape of the a) Cu^{+}, b) $Ag^{+,}$ c) Au^{+}, d) Th^{+}.

Figure S2 presents the shape of a) the oblique hat function Eq. 2, b) the Gaussian Eq. 3, and c) the convolued function Eq. 4, for a specific choice of parameters: *c=0 amu, w=0.5 amu, W=1.5 amu, α=0.2 and r=1.*

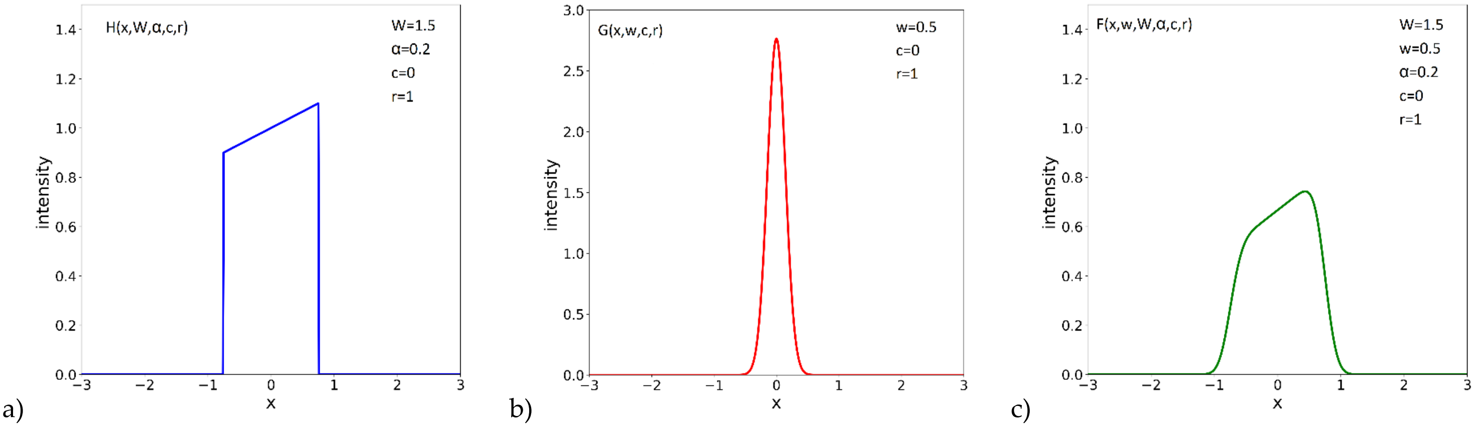

Figure S2. The oblique hat function H (W=1.5, a=0.3, c=0, r=1) the Gaussian G (w=0.5, c=0, r=1) and the "peak model function" F resulted from the convolution (w=0.5, W=1.5, a=0.3, c=0, r=1

The variations of the peak shape parameters, i.e., the Gaussian w, oblique hat width W and obliquity α, upon $(m/z)_{real}$ value for the well identified peaks are presented in Figure S3. The linear function Eq. 8 (main text) is used to determine the parameter's values in any other *m/z* positions and the linear regresion parameters, namely a and b, are shown for each case in the legends of the Figures. The error bars from each figure represent the fit parameters variations, calculated as the diagonal values of the estimated covariance matrix of optimal parameters, as resulted from the fit of the individual peaks. The variation of a parameter indicates how much its value could change from the best-fit value such that the quality of the fit is not strongly affected. We computed the variance as a percentage of the parameter's value and obtain that w and α can vary by almost 25% of their value while W by less than 0.2%. Small variation in the center parameter (~0.01%) strongly affected the quality of the fit.

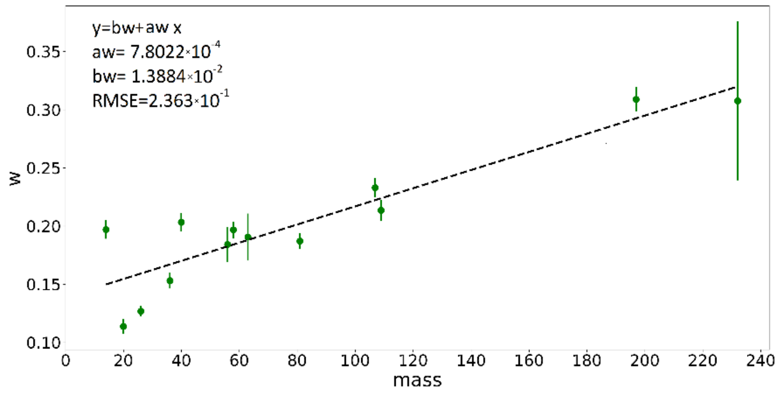

a)

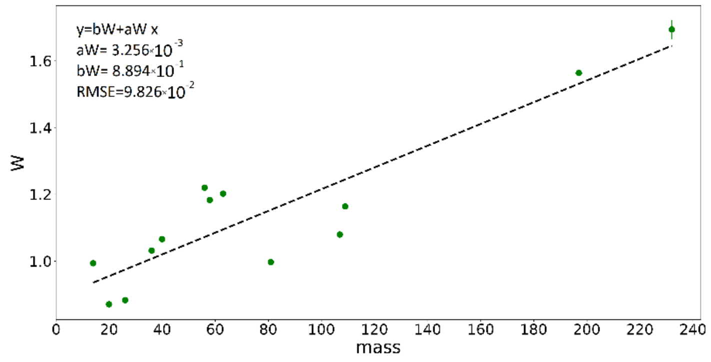

b)

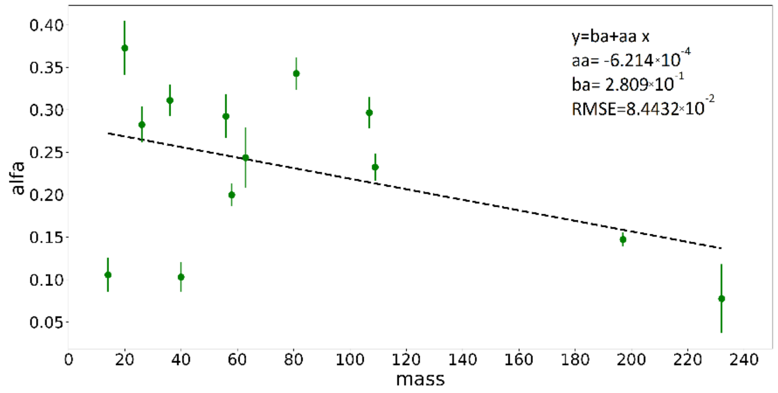

c)

Figure S3. The $(m/z)_{real}$ dependence of the a) Gaussian width w, b) the oblique hat width W and c) obliquity α, together with the linear fit parameters and the RMSE value.

The detailed results presenting the computed abundance (CA) of the W isotopes from the multielement (ME) and W plasma in Ar are shown in Table S1. The fitting of the experimental data is performed using 5 free parameters per peak or only one free parameter (area of the peak) with the shape and center parameters fixed by the calibration curved. The NRMSE value (Eq. 7) of each fit is presented in Table S1, together with the relative error of the abundance of each peak and the mean error.

**Isotope**	**NA**	**CA-Fit 5 params. [1] ME**	$\frac{\lvert NA - CA \rvert}{NA} \times 100$	**CA-Fit 1 params. [1] ME**	$\frac{\lvert NA - CA \rvert}{NA} \times 100$	**CA-Fit 5 params. [1] W/Ar**	$\frac{\lvert NA - CA \rvert}{NA} \times 100$	**CA-Fit 1 params. [1] W/Ar**	$\frac{\lvert NA - CA \rvert}{NA} \times 100$
^{182}W	26.5	27.33	3.31	27.38	3.32	26.36	0.52	26.5	0
^{183}W	14.31	15.73	9.92	13.66	4.54	16.12	12.64	15.73	9.92
^{184}W	30.64	28.88	5.74	30.86	0.71	29.54	3.59	30.05	1.92
^{186}W	28.43	28.04	1.37	28.08	1.23	27.96	1.65	27.70	2.56
$\sigma(\%) = \frac{1}{4}\sum\frac{\lvert NA-CA \rvert}{NA}\times 100$		-	5.04	-	2.45	-	4.60	-	3.60
**NRMSE (%)**		8.61	-	10.174	-	8.417	-	19.36	-

[1] Per peak

Table S1. The values of the natural abundance (NA) of W isotopes and the computed abundance (CA) obtained from the fit, together with the NRMSE, and the error calculated as the absolute value of the difference between NA and CA, divided by NA and summed over all peaks.
